# Human Immunodeficiency Virus (HIV) Masquerading as Myopathy and Rhabdomyolysis

**DOI:** 10.7759/cureus.14559

**Published:** 2021-04-19

**Authors:** Kushal Ranabhat, Smit Deliwala, MurtazaShabbir Hussain, Tarek Haykal, Ghassan Bachuwa

**Affiliations:** 1 Internal Medicine, Michigan State University at Hurley Medical Center, Flint, USA; 2 Division of Oncology, Department of Internal Medicine, Duke University, Durham, USA

**Keywords:** hiv, rhabdomyolysis, myopathy, creatinine kinase, aids

## Abstract

Human immunodeficiency virus (HIV) characteristically presents as a mononucleosis-like prodrome; rhabdomyolysis as a sole manifestation remains a rare finding from infection to seroconversion. A young male with a vague sexual history presented with myopathy progressing rapidly to rhabdomyolysis and renal failure. Acute HIV rarely presents with classic features, and rhabdomyolysis seems to manifest more in younger patients. Our case also demonstrates the importance of keeping a strong suspicion for HIV in the right setting despite false-negative results in the pre-seroconversion phase. The variability in HIV presentation and stigma of sexual history taking represents a diagnostic challenge. The astute clinician must be privy to these peculiarities to formulate a prompt diagnosis.

## Introduction

Primary HIV infection (PHI) denotes the period of infection to seroconversion, during which rapid viral load accumulates, prompting viral antibodies from the host immune system [1]. Classically, seroconversion mimics a mononucleosis-like syndrome that occurs two to six weeks after exposure, but alternative presentations have been reported. Acute rhabdomyolysis is commonly caused by drug interactions, trauma, electrolyte derangements, toxins, and infections, releasing harmful contents into the bloodstream [2]. Infrequently, rhabdomyolysis has been associated with PHI, but few case reports describe it as the sole presentation of an acute infection. HIV-associated myopathy presenting with rhabdomyolysis and complicated by renal manifestation can have life-threatening presentations, requiring prompt recognition and initiation of antiretroviral therapy [3]. We report a patient whose sole presentation of PHI was rhabdomyolysis, but initial history taking and screening were inadequate. This case highlights the importance of diverse PHI presenting symptoms, obtaining an accurate history to identify sexually transmitted infections (STI), and the importance of gender-neutral sexual history tools to lessen delays in urgent antiretroviral therapy.

## Case presentation

A previously healthy 21-year-old male presented with evolving nausea, vomiting, and abdominal pain over the preceding week. Initial workup at an urgent care was unremarkable for hepatitis, Epstein-Bar virus (EBV), and rapid HIV (antigen/antibody) 1 & 2, and no risk factors were identified. He was recommended conservative measures and sent home on close follow-up, however, his symptoms worsened to generalized weakness, cramping pains, fatigue, malaise, and fever, prompting a visit to the emergency department (ED) three days later. On arrival, he was hypotensive to 77/46 mm/hg, normocardic, afebrile, appearing weak and ill on physical exam. He was tender to palpation in both calves and muscle strength was 2-3/5, and no accompanying neurological deficits were seen including sensory testing. Laboratory workup revealed leukopenia to 2.4K/UL, thrombocytopenia 91K/UL, creatinine kinase (CK) 9519 U/L, lactate dehydrogenase 856 U/L, and aldolase 39.4 U/L. He has aggressively hydrated with lactated ringers. A subsequent reduction in CK levels was seen. A comprehensive workup was negative for infectious, autoimmune, or endocrine causes (Table 1). Keeping his age, presentation, and local rates, we strongly suspected HIV. The state of Michigan requires patient consent before testing, and after explaining our rationale, the patient provided consent. A 4th generation antigen and antibody test was positive with a polymerase chain reaction (PCR) assay growing 10,000,000 copies/ml, with positive HIV-1 seroconversion on a differentiation assay. Initial speciation of lymphocytes revealed a CD4 count of 195 cell/UL. Upon hearing the diagnosis, our patient admitted a bisexual history and that one of his partners had mentioned a possible HIV exposure in the past, although testing was never completed. An infectious disease consult recommended initiating bictegravir, emtricitabine, and tenofovir. His clinical course stabilized, and he was discharged on close follow-up. He had undetectable viral levels with a CD4 count of 673 cell/uL at his one-month follow-up and continued to have undetectable levels with a CD4 count of 617 cell/uL at his one-year follow-up.

**Table 1:** Laboratory investigations used CMV - Cytomegalovirus; EBV - Epstein-Barr virus; VCA - Viral capsid antigen; PCR: Polymerase chain reaction; ENA - Extractable nuclear antigen; RNP - Ribonucleic peptide

Investigation	Result
Beta Hydroxy Butyrate	<2 mg/dL
Creatinine kinase (CK)	9519 u/L
Creatinine	0.7 mg/dl
Urinalysis	Blood +1, Protein +1, yellow
Lactate	1.9 mmol/l
Aspartate Transaminase (AST)	719 U/L
Alanine Transaminase (ALT)	346 U/L
Haptoglobin	29.3 mg/dl
Beta Hydroxy Butyrate (b-OH)	<2.0 mg/dl
Thyroid Stimulating Hormone (TSH)	4.77 UIU/ML
Aldolase	39.4 u/l
Hepatitis panel – Hepatitis B surface antigen, Hepatitis B core antibody IgM, Hepatitis C antibody, and Hepatitis A IgM antibody	Negative
CMV IgM/IgG Ab EIA	Negative
EBV VCA Antibody panel	Negative
Syphilis	Non-reactive
Urine gonorrhea/chlamydia PCR	Negative
Blood cultures	No growth after 6 days
Cryptococcal antigen	Negative
Drug screen – opiates, marijuana, amphetamine, cocaine, benzodiazepines, ethanol, phencyclidine, methadone, and oxycodone	Negative
Salicylate/Acetaminophen levels	Negative
Antinuclear antibodies	Negative
Rheumatoid factor titer, Anti-Jo 1 IgG, and Anti-smooth muscle antibody	Negative
Plasma cortisol	14 ug/dl
ENA to Smith (sm) antibody, RNP antibody, SSA (Ro) antibody, SSB (La) antibody, and Antimitochondrial antibody	Negative
Erythrocyte sedimentation rate	6 mm/hr
Lactate dehydrogenase	856 u/l

## Discussion

PHI is a hyper-infectious state, and its prompt identification and treatment can prevent further transmission and reduce its inflammatory condition. With the appropriate sexual history and typical symptoms, suspicion for PHI can warrant pertinent testing. However, rhabdomyolysis is an exceedingly rare presentation of PHI, with an incidence rate of 0.943% [4]. Broadly, HIV myopathy can be classified as 1) HIV-associated rhabdomyolysis, 2) HIV therapy-induced rhabdomyolysis, and 3) opportunistic infection provoked myopathy in acquired immunodeficiency syndrome (AIDS), with the latter two more frequently reported. PHI presenting as rhabdomyolysis was first reported in 1989, and a literature review has been summarized in Table 2. A retrospective study demonstrated that lower viral loads were more consistent with anti-retroviral-induced myopathy. In our patient, HIV myopathy likely resulted from direct viral replication within the muscle leading to rhabdomyolysis and widespread fatigue [5]. Moreover, severe HIV presentation incited immunosuppression may predispose to acute myositis, as observed from our patient’s AIDS defined CD4 count of 195 cell/UL [6]. Overall, HIV incidence has decreased, except in sexually active homosexual men, at approximately 10,000 infections among African American men who have sex with men (MSM), fitting our patient’s demographic [7]. Advanced 4th generation HIV antigen and antibody combination assay is gradually replacing point-of-care rapid HIV testing as the primary diagnostic, as the latter has false negatives in the acute phase [2]. Nevertheless, up to 75% of PHI can remain undiagnosed due to the diversity of clinical presentation [8], demonstrating the importance of thorough sexual history taking or a high index of suspicion, similar to our patient encounter. HIV rhabdomyolysis is confirmed via CK elevation and the proper seroconversion findings. A biopsy is rarely required. The full extent of our patient’s sexual history was elicited only after confirmatory combination assay testing. Physicians admitted a need for further education and training regarding sexual history taking [9]. Implementing a standardized, time-efficient, and gender-neutral sexual history-taking tool may mitigate stigma with at-risk populations. Moreover, in patients with appropriate risk factors, combination testing after a negative rapid HIV test may lessen the delays in viral detection and reduce lifetime HIV costs [10]. Physicians should utilize sexual history-taking tools in addition to staying vigilant of PHI in a patient with unanticipated muscle pains and elevated CK levels, especially in younger individuals.

**Table 2:** Previously published case reports of rhabdomyolysis as the singular manifestation of primary HIV (PHI) Age in years; HIViral load in copies/mL; CD4 in cells/uL; Creatinine in mg/dL; Lactate in U/L; AST and ALT in U/L Dx: Diagnosis; N/A: Not applicable; CK: Creatinine kinase; SCrt: Serum Creatinine; F/U: Follow-up; AST: Aspartate transaminase; ALT: Alanine transaminase; CPK: Creatinine phosphokinase; ↑: Increased.

Author/Year	Age	Sex	HIV+ duration	HIV Stage	Viral Load	CD4	SCrt	CK	Lactate	AST	ALT	F/U CPK	Outcome
Prabahar et al., 2008 [11]	42	M	New Dx	Acute	638,000	436	6.8	278,000	N/A	3,640	419	623	Recovery
Ferrada et al., 2015 [12]	19	M	New Dx	Acute	463,331	436	8.3	4,000	N/A	N/A	N/A	4,000	Recovery
Mahe et al., 1989 [13]	18	M	New Dx	Acute	N/A	304	1.3	5,750	882	165	N/A	106	Recovery
Babiker et al., 2015 [14]	61	M	New Dx	Acute	606,183	200	0.8	10,055	N/A	644	1,121	150	Recovery
Hernandez-Munoz et al., 2013 [15]	51	M	New Dx	Acute	150,000	N/A	13.0	104,000	N/A	↑	↑	N/A	N/A
Neves et al., 1991 [16]	24	F	New Dx	Acute	N/A	N/A	0.5	2,359	↑	N/A	N/A	N/A	Recovery
Guillaume et al., 1995 [6]	42	M	New Dx	Acute	N/A	N/A	1.9	63,800	2820	1,480	461	N/A	Recovery
Rastegar et al., 2001 [17]	51	M	New Dx	Acute	750,000	515	1.6	32,720	N/A	875	236	2,700	Recovery
McDonagh and Holman, 2003 [18]	33	M	New Dx	Acute	750,000	N/A	0.8	18,840	N/A	321	98	N/A	Recovery
Delo et al., 2006 [2]	46	M	New Dx	Acute	750,000	N/A	0.8	4,718	N/A	122	40	1,891	Recovery
Pano-Pardo et al., 2008 [19]	19	F	New Dx	Acute	100,000	264	2.3	10,681	N/A	153	103	N/A	Recovery
Douvoyiannis and Litman, 2009 [1]	20	M	New Dx	Acute	500,000	140	1.6	2,968	N/A	266	254	N/A	Recovery
Halperin et al., 2012 [8]	33	F	New Dx	Acute	1,500,000	410	N/A	36,725	N/A	N/A	N/A	N/A	Recovery
Bhargava et al., 2016 [3]	49	F	New Dx	Acute	172,828	13	6.7	54,275	N/A	N/A	N/A	N/A	Recovery
Haddiya et al., 2016 [20]	45	M	New Dx	Acute	N/A	200	2.0	1,900	N/A	N/A	N/A	N/A	Death
Noe et al., 2018 [5]	24	M	New Dx	Acute	10,000,000	170	5.7	200,000	N/A	N/A	N/A	N/A	Recovery

## Conclusions

Primary HIV infection (PHI) has a wide range of clinical presentations; clinicians should be attentive to rhabdomyolysis as a lone manifestation. This allows for prompt treatment and counseling. The employment of a standardized sexual history-taking tool can curtail stigma in vulnerable patients lowering the number of undiagnosed individuals in the community. Strong clinical suspicion justifies combination assay testing immediately after rapid HIV screening, decreasing delays in diagnosis and treatment.
